# Mathematical complexities in radionuclide metabolic modelling: a review of ordinary differential equation kinetics solvers in biokinetic modelling

**DOI:** 10.1088/1361-6498/ad270d

**Published:** 2024-05-21

**Authors:** Emmanuel Matey Mate-Kole, Shaheen Azim Dewji

**Affiliations:** 1 Nuclear and Radiological Engineering and Medical Physics Programs, Georgia Institute of Technology, Atlanta, GA, United States of America

**Keywords:** ordinary differential equations, biokinetic models, internal dosimetry, ODE solvers, dose reconstruction, ICRP, PBPK

## Abstract

Biokinetic models have been employed in internal dosimetry (ID) to model the human body’s time-dependent retention and excretion of radionuclides. Consequently, biokinetic models have become instrumental in modelling the body burden from biological processes from internalized radionuclides for prospective and retrospective dose assessment. Solutions to biokinetic equations have been modelled as a system of coupled ordinary differential equations (ODEs) representing the time-dependent distribution of materials deposited within the body. In parallel, several mathematical algorithms were developed for solving general kinetic problems, upon which biokinetic solution tools were constructed. This paper provides a comprehensive review of mathematical solving methods adopted by some known internal dose computer codes for modelling the distribution and dosimetry for internal emitters, highlighting the mathematical frameworks, capabilities, and limitations. Further discussion details the mathematical underpinnings of biokinetic solutions in a unique approach paralleling advancements in ID. The capabilities of available mathematical solvers in computational systems were also emphasized. A survey of ODE forms, methods, and solvers was conducted to highlight capabilities for advancing the utilization of modern toolkits in ID. This review is the first of its kind in framing the development of biokinetic solving methods as the juxtaposition of mathematical solving schemes and computational capabilities, highlighting the evolution in biokinetic solving for radiation dose assessment.

## 1. Introduction

**Figure 1.**
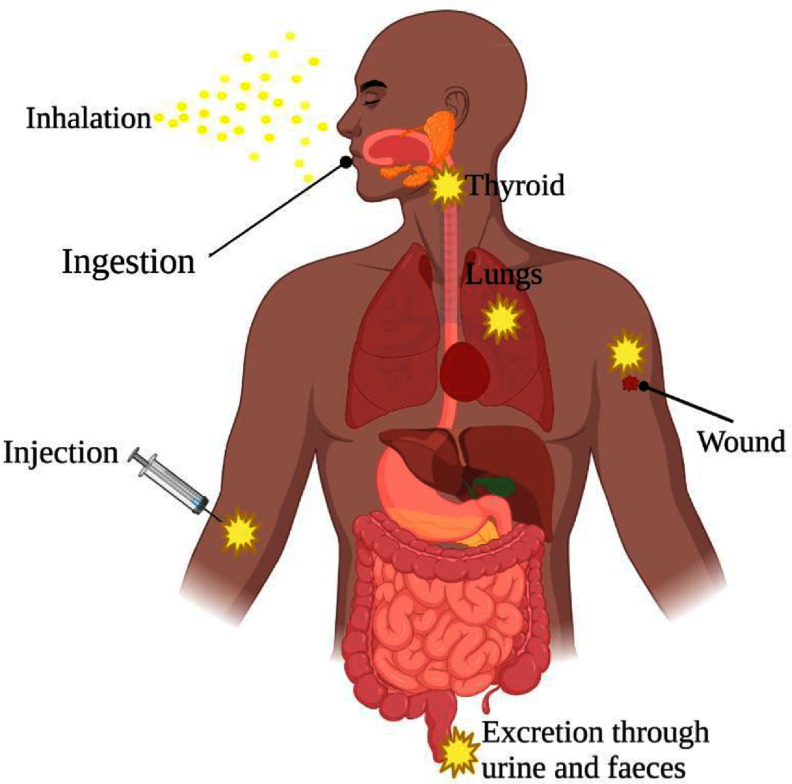
Primary internal exposure pathways. This figure has been created with BioRender.com. (BioRender 2020).

Internal dosimetry deals with the determination of radionuclide distribution in the tissues/organs within the body (Zanzonico 2000). Radionuclides can be internalized through inhalation, ingestion, and wound dosimetry pathways. Internal exposure through these pathways affects multiple systems, as illustrated in figure 1, which include inhalation, where intake occurs through the respiratory tract and uptake systemically occurs through the lungs; and ingestion, where intake occurs through the mouth, and where absorption and systemic uptake occurs through the alimentary tract system.

Due to the inability to directly measure the radionuclide content in specific organs in the body, internal dosimetry (ID) relies heavily on complex mathematical formalism coined as biokinetic models (Bertelli *et al* 1997) with three main objectives (Potter 2004): (1) to provide timely feedback on workplace control; (2) to initiate medical intervention; and (3) to show compliance with regulations.

Fundamentally, the term *biokinetic* is derived from the Greek word *bio* (life) and *kinetic* (transport) (Li 2018). Thus, biokinetic models have evolved to represent the movement of radionuclides as a compartmental representation of the human body by which retention and excretion are mathematically modelled as a system of coupled ordinary differential equations (ODEs) for overall dose assessment. It is therefore critical to acknowledge that due to the complexity of the metabolic pathways, and differences in chemical and physical properties of incorporated radionuclides, multiple biokinetic models must be constructed based on the specific internal exposure pathways relevant to the incorporated element.

The biokinetic model, as a dynamic system, can be approached as a system of mass balance equations describing the flow of materials in and out of the organs/tissues of the body. For modelling purposes, the organs/tissues as single components may be characterized in terms of multiple compartments. For example, the biokinetic model of the liver for a lanthanide element is divided into Liver 1 (short-term) and Liver 2 (long-term) compartments (ICRP 2019). The transfer of materials in and out of a compartment (including recycling back into compartments) is represented by transfer coefficients, which quantify the fractional transfer of contents in and out of an organ per unit time. It is worth noting that although transfers between the compartments are often represented by first-order kinetics, it is not a one-size-fits-all approximation. Studies have shown that with an increase in concentration of vinyl chloride above saturation, for example, its clearance follows zero-order kinetics (Hefner *et al* 1975, World Health Organization 1999). In mathematical terms, this system is framed as a series of ODEs. To the mathematician, any entity that changes is a variable, and the rate of change of that variable is a derivative (Tenenbaum and Pollard 1985). Differential equations model the variation of one parameter with respect to another. Such mathematical models containing only ordinary derivatives of one or more unknown function(s) with respect to an independent variable are known as ODEs (Zill 2018). ODEs provide a governing framework for how a given state variable changes over an infinitesimal interval. Generally, the body’s dynamic material exchanges are governed by standard mass balance equations, describing the inflow and outflow in/out of a designated compartment (Anderson 1983). The standard mass balance, which models the rate of change of mass in/out of a compartment, conforms to an ODE and thus warrants its applicability for modelling dynamic systems for various applications such as analysis of the ecosystem, chemical reactions studies, drug kinetics in pharmacology, climate modelling, and studies of metabolic systems including biokinetic modelling (Anderson 1983, Aro 1996, Postawa *et al* 2020).

An ODE can be categorized as non-stiff or stiff, whereby non-stiff ODE systems evolve simultaneously, while stiff systems are considered to be systems for which the solutions include slowly and rapidly varying components (Byrne and Hindmarsh 1987, Aro 1996). Due to the highly dynamic form and complexity of biokinetic models, the problems posed by biokinetic models are mostly considered stiff and, as a result, require a careful selection of solving methods, whether analytically or numerically. These methods are scripted as solvers or algebraic algorithms, which are then packaged into computer codes for expedited calculations. Biokinetic models are adopted to estimate the dose from internalized radionuclides for radiation protection purposes, which are heavily reliant on mathematical frameworks, predominantly describing the biodistribution of materials in the body. With this level of conformity, the computer codes and algorithms are leveraged by ID experts for an expedited radiation dose assessment without sacrificing accuracy.

In this review, the mathematical conception of biokinetic models leading to the calculation of internal dose is surveyed. A general overview of biokinetic models is first introduced, followed by a discussion of their evolution and increasing complexity, mathematical solving frameworks, and their computational implentation. Ultimately, several internal dose computer codes focusing on high-level scripted procedural solving methods are presented, and in an expanded discussion, the mathematical complexities and formulations are discussed. Given the continuous updates and improvements of biokinetic models and computational tools, this review uniquely provides a comprehensive analysis of biokinetic solving methods and base knowledge for understanding the computational demands, schemes, and implementations for biokinetic modelling.

## 2. Mathematical conception of biokinetic models

### 2.1. Biokinetic modelling in radiation protection

Prior to the mid-1960s, knowledge of the quantification of internally incorporated radionuclides was limited (Stather 2004). However, the establishment of organizations, including the International Commission on Radiological Protection (ICRP) and the National Council on Radiation Protection and Measurements (NCRP), have advanced the knowledge of radiation protection through recommendations and guidance. Notwithstanding the variety of organizations with an interest in this area, the ICRP continues to serve as the preeminent authority in the recommendation of biokinetic models. These biokinetic models have been widely adopted for prospective and retrospective radiological protection applications, relying on a multitude of individual and element-specific studies (ICRP 1959, 1972, 1994, 2006, 2015, NCRP 1997, NCRP 2006, Li 2018).

**Figure 2.**
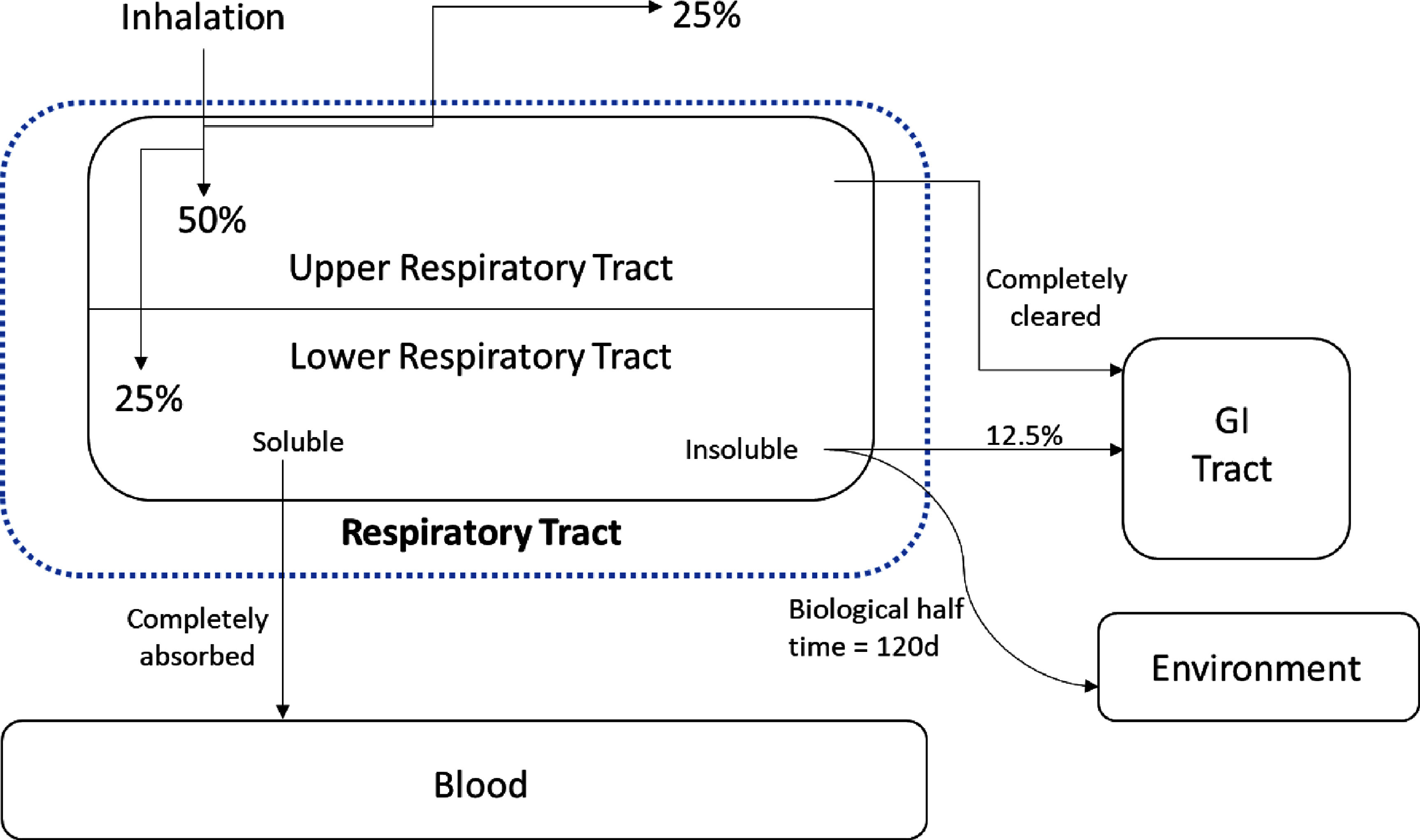
An illustration of a simple linear compartmental model of the respiratory tract described by the ICRP Publication 2 (ICRP 1959).

To quantitatively estimate internal dose, the knowledge of the biokinetics of the incorporated radionuclide must be addressed first. Fundamentally, biokinetic models adopt a compartment-based approach to reflect the physiology of the system under study and to represent the physical location of substances within that system (Vicini *et al* 2008). This approach makes it suitable to mathematically represent biokinetic models as a system of ODEs (Li 2018). According to ICRP Publication 30 (1979), the loss of radionuclides from the compartments are described by first-order kinetics with constant coefficients except for alkaline earth metals, for which the metabolic behaviour is not entirely governed by first order rate constants. Thus, ICRP Publication 30 highlighted an alternative approach for modelling alkaline earth metals. The challenges in framing the equations for any radionuclide chain member were later addressed (Polig 2001, Fell *et al* 2007). The exact solution of the system of equations governing the metabolic models (solved without feedback consideration) has been investigated in ICRP Publication 2 (figure 2 is a simple linear compartment model of the respiratory tract) (ICRP 1979), thus building the ground-zero knowledge and capabilities to solve for the radionuclide distribution under specific boundary conditions. Since then, more complex biokinetic models have been developed, incorporating variable transfer rates and recycling of materials between compartments (Leggett *et al* 1993). Consequently, the foundational system of equations describing biodistribution, which is needed for internal dose estimation, remains the same. The general form of the rate of exchange of the radionuclide activity is represented by a set of first-order linear differential equation in equation (1) (ICRP 2015, Issa and Serge 2021):

\begin{equation*}\frac{{{\text{d}}{A_{i,j}}\left( t \right)}}{{{\text{d}}t}} = \sum\limits_{\begin{array}{*{20}{c}} {k = 1} \\ {k \ne j} \end{array}}^M {A_{i,k}}{\lambda _{i,k,j}} - {A_{i,j}}\left[ {\sum\limits_{\begin{array}{*{20}{c}} {k = 1} \\ {k \ne j} \end{array}}^M {\lambda _{i,{\text{j}},{\text{k}}}} + \lambda _i^P} \right] + \sum\limits_{k = 1}^{i - 1} {A_{k,j}}{\beta _{k,i}}\lambda _{i{ }}^P\end{equation*}

where *M* is the number of compartments describing the kinetics; ${\lambda _{i,j,k}}$ is the fractional transfer rate of chain member *i* from compartment *j* (donor compartment) to compartment *k* (receiving compartment) in the biokinetic model; $\lambda _i^P$ is the physical decay constant of chain member *i;* and ${\beta _{k,i}}$ is the fraction of decays of chain member *k* forming *i.*

### 2.2. Decorporation modelling

Over the past decade, radiation countermeasures have become an essential focus for mitigating and treating radiation injuries, forming the basis of decorporation therapy (Rosen *et al* 2015, Singh and Seed 2017). Decorporation therapy utilizes chemical compounds (chelation agents) to accelerate the body’s clearance of incorporated radionuclides/metals (Dumit *et al* 2019). For commercial applications of these chemicals, industrial guidelines require that the efficacies of these drugs are demonstrated, which are usually investigated through computational modeling (Miller *et al* 2012). The administration of decorporation agents adds to the complexity of the mathematical representation of the biokinetic models described. In contrast to equation (1), the mathematical description of the decorporation process must additionally consider the chemistry of the incorporated radionuclide/metal under physiological conditions. Several mathematical approaches for modelling decorporation therapy have been discussed in the literature (Hall *et al* 1978, LaBone 1994, Fritsch *et al* 2007, James *et al* 2007). To illustrate the basic idea of the mathematical formalism, the coordinated network for radiation dosimetry (CONRAD) approach (Breustedt *et al* 2009) is discussed in this review. The decorporation process is modelled as second-order kinetics to represent the competing reactions of the incorporated metal and the chelation agent in the body (Miller *et al* 2018), thus, introducing nonlinearity in the differential equations (DEs) to solve. According to the CONRAD approach, the biokinetics of the incorporated metal (plutonium in the CONRAD study) and the injected decorporation agent (diethylenetriamine pentaacetate [DTPA] in the CONRAD study) are treated as independent compartmental models, which relate to an appropriate mathematical representation of the decorporation process.

The mathematical system governing the biokinetic modelling of decorporation agents comprises three matrices: *x* (compartments representing the biokinetics of the decorporation agent, as given in equation (2)); *y* (compartments representing the biokinetics of the incorporated metal only, as given in equation (3)); and *z* (the compartments indicating the chemical complexes of the metal and the decorporation agent, as given in equation (4)). The system of equations can be represented as follows (Breustedt *et al* 2009, 2010):

\begin{equation*}\frac{{{\text{d}}{x_i}}}{{{\text{d}}t}} = - \sum\limits_{j = 1}^n {k_{ij}}{x_i} + \sum\limits_{j = 1}^n {k_{ji}}{x_j} - {\text{CR}}.f\left( {{x_i},{y_i}} \right)\end{equation*}

\begin{equation*}\frac{{{\text{d}}{y_i}}}{{{\text{d}}t}} = - \sum\limits_{j = 1}^n {k_{ij}}{y_i} + \sum\limits_{j = 1}^n {k_{ji}}{y_j} - {\text{CR}}.f\left( {{x_i},{y_i}} \right)\end{equation*}

\begin{equation*}\frac{{{\text{d}}{z_i}}}{{{\text{d}}t}} = - \sum\limits_{j = 1}^n {k_{ij}}{z_i} + \sum\limits_{j = 1}^n {k_{ji}}{z_j} + {\text{CR}}.f\left( {{x_i},{y_i}} \right)\end{equation*}

where *n* is the number of compartments; *i* and *j* are the compartments indices; ${k_{ij}}$ and ${k_{ji}}$ describe the biokinetic transport of materials from and to each compartment; CR is the chelation rate for the chelation process; and $f\left( {{x_i},{y_i}} \right)$ is a function that describes the chelation process—thus, the function is normally characterized by the product of *x* and *y* (Breustedt *et al* 2009). This model, however, is said to be not fully realistic and did not fully incorporate chemical speciation. Although the CONRAD approach utilizes second-order kinetics for the chelation process, a study conducted by Konzen and Brey (2015) revised the radionuclide-chelation (specifically plutonium-DTPA) biokinetic model proposed by Breustedt *et al* (2009) to include four transitional state compartments intended to describe the chelation process to utilize first-order kinetics. According to Konzen and Brey (2015), the revised model is to provide additional insights into the usage of DTPA and its therapeutic benefits.

### 2.3. Translation to ID software

The complexities resulting from a system of hundreds of ODEs in some cases, including recycling, become cumbersome when approached through manual solving or by some classical means. These complexities motivated the development and introduction of internal dose programs/computer codes for mainly radiation protection and medical applications for quick and easy calculation turnaround and reproducible results. These programs solve the system of ODEs using appropriate mathematical functions or methods depending on the difficulty of the problem sets. The approach was dictated by whether the biokinetic model is simple or complex based on the number of parameters involved, whether it employs a recycling approach and whether it accounts for chemical and biological transformations due to physiological processes. To this effort, several computer programs were written to perform the task of complex ODE solving based on the existing mathematical and computational capabilities representative of the era. Most of these computer codes are usually coupled with a computational module for computing the mean absorbed dose received by the target organ from an incorporated radionuclide for the purpose of internal dose assessment. The mean absorbed dose module can be either an external computational module or as an inherent subroutine/function script in the program. Table 1 outlines a list of documented internal dose codes and their respective ODE solvers/methods, for which expanded discussions are carried out in the subsequent section.

**Table 1.** Survey of computational codes and programs for modelling the distribution of and dosimetry of internal emitters.

Internal dose code	ODE solver/Methods	Programming platform
INDOS (Killough and Rohwer 1974)	Exponential function, power function or combination of both methods	FORTRAN IV
TIMED (Watson *et al* 1976)	The Gear package: Implicit Adams method, and backward differentiation formula (BDF) methods	Available in either of the following: IBM System/360, System/370 in FORTRAN IV, Assembler language for IBM System/360
INREM-II (Killough *et al* 1978)	Linear combination of decaying exponentials	FORTRAN IV for IBM-360 or 370
AGEDOS (Leggett *et al* 1984)	Linear combination of decaying exponentials	FORTRAN IV for IBM 3033
DIFSOL (Killough and Eckerman 1984)	Eigenvalue method	FORTRAN IV and translated into BASIC
CINDY (Strenge *et al* 1990)	ODEPACK solver: LSODES—a backwards differentiation formulation, based on the multistep methods	Standard FORTRAN 77 with Lahey compiler
GENMOD (Richardson and Dunford 1998)	Numerical method: CVODE	Mainframe written in FORTRAN,PC version in C and FORTRAN and updated version for ICRP 60/66 models was written in C++
IMBA (Birchall *et al* 1998)	Analytical from Birchall (1985) study	Subroutine-based algorithms/Visual basic and inputs from ASCII data files
SAAM II (Barrett *et al* 1998)	Rosenbrock integrator (semi-implicit method),Forward-integration Runge–Kutta method, and Padé integrator (Padé approximation)	C++
INDOSE (Silverman 2002)	Numerically with solver LSODES specifically adapted to sparse matrices	FORTRAN90
MONDAL3 (Ishigure *et al* 2004)	Numerical: Runge–Kutta method	Microsoft visual basic for interface program
OLINDA/EXM *(A rewritten version of MIRDOSE)* (Stabin *et al* 2005)	Sum of exponentials	Java (Sun microsystems)
BIOKMOD (Sanchez 2005)	Analytical: matrix exponentials Laplace transforms	MATHEMATICA
DCAL (Eckerman *et al* 2006)	Approximated the first-order kinetics in an isolated system iteratively proposed by Eckerman *et al* (1992)	FORTRAN with interactive interface written in Professional BASIC
PLEIADES (Fell *et al* 2007)	Eigenvalue method	NAG Fortran library
IMIE (Berkovski *et al* 2007)	Numerical: Runge–Kutta Method	Unknown but distributed on CD-ROM for installation
IMPDOS (Miller *et al* 2008)	DLSODES: Livermore solver for ODEs with general sparse Jacobian matrix	FORTRAN 77
AIDE (Bertelli *et al* 2008)	Eigenvalue method	Routine-based: FORTRAN
IDode (Miller *et al* 2012, 2019)	DLSODES: Livermore solver for ODEs with general sparse Jacobian matrix	Fortran. Graphical user interface (GUI) for IDose was written in Visual Basic 6 (VB6) by Luiz Bertelli. Modifications were made by Guthrie Miller to run IDode.exe—this has been compiled using the Intel Fortran compiler
J-LSODE (Manabe *et al* 2019)	Numerical: LSODE solver	Java
TAURUS (UK Health Security Agency 2020)	Provides a graphical user interface (GUI) for PLEIADES. PLEIADES code implements eigenvalue method	GUI was built using the Winteracter Portable Fortran user interface. The graphics toolset was built by Interactive Software Services Ltd.
IDAC-Bio (Andersson *et al* 2022)	Stepwise numerical integration	MATLAB

NB: The table outlines internal dose computer programs with identified solvers for kinetics.

Prior to 2005, most of the earlier solvers were developed based on simpler biokinetic models (in most instances, complex biokinetic models were not yet available). As complex models became available and desktop computers became widely accessible, updated versions of the computer codes or a completely new code were developed to accommodate recent metabolic updates (Birchall *et al* 2005). For example, a computer program for calculating cumulated radionuclide activity in organs of the human body at a given time post deposition named TIMED was described by Watson *et al* (1976). According to Watson *et al* (1976), TIMED as a dosimetry code is executable on the IBM System/360 or System/370 machines. Thus, it had limited accessibility. Consequently, considering exposure scenarios and region-specific source terms warranted the construction of new computer programs (Manabe *et al* 2019). Some of these internal dose computer codes entailed more than one mathematical solving methodology scripted as solvers, each having specific strengths and limitations for tackling specific subsets of metabolic systems. Also, different flavours of the codes were written in different programming languages, such as Mathematica (Wolfram Research Inc 2022), FORTRAN (Kedward *et al* 2022), and Java (Arnold *et al* 2005), based on the needs of the developer, such as but not limited to the following:

1. The need for the program to have the ability to execute on various computer platforms (Manabe *et al* 2019),
2. Computational speed, memory constraints (Richardson and Dunford 1998), and difficulties in migration onto newer computer operating systems (Stabin *et al* 2005).

Kinetic models are an invaluable tool for understanding the dynamic response of biological systems. However, large-scale applications of these models are largely limited by the availability and robustness of computational tools (Weilandt *et al* 2023). In the remainder of this paper, the use of ODEs as a mathematical solving tool will be discussed. A review of existing and evolving solvers and solving methods will be conducted, with a specific focus on expanding discussions concerning the solution methods employed for modelling the biodistribution of internal emitters.

## 3. Overview of forms of ODEs

The system of equations holds significance in ID, as it offers researchers and practitioners the flexibility to decompose dynamic exchanges within the body into a finite number of components. This allows for a mathematical representation of specific biochemical processes. The eventual implementation of this system contributes to a more comprehensive understanding of ID. Once physiological processes are mathematically represented, the framework becomes more clearly defined to follow material exchanges. This section first provides the framework governing underlying mathematical models and outlines ODE forms and methods. Overall, the section summarizes the foundational elements in the mathematical methodologies appropriate for compartmental analysis by emphasizing their respective strengths and weaknesses.

### 3.1. ODE fundamentals

As a desirable approach, the behaviour of some real-life phenomena is primarily represented by mathematical equations.

**Figure 3.**
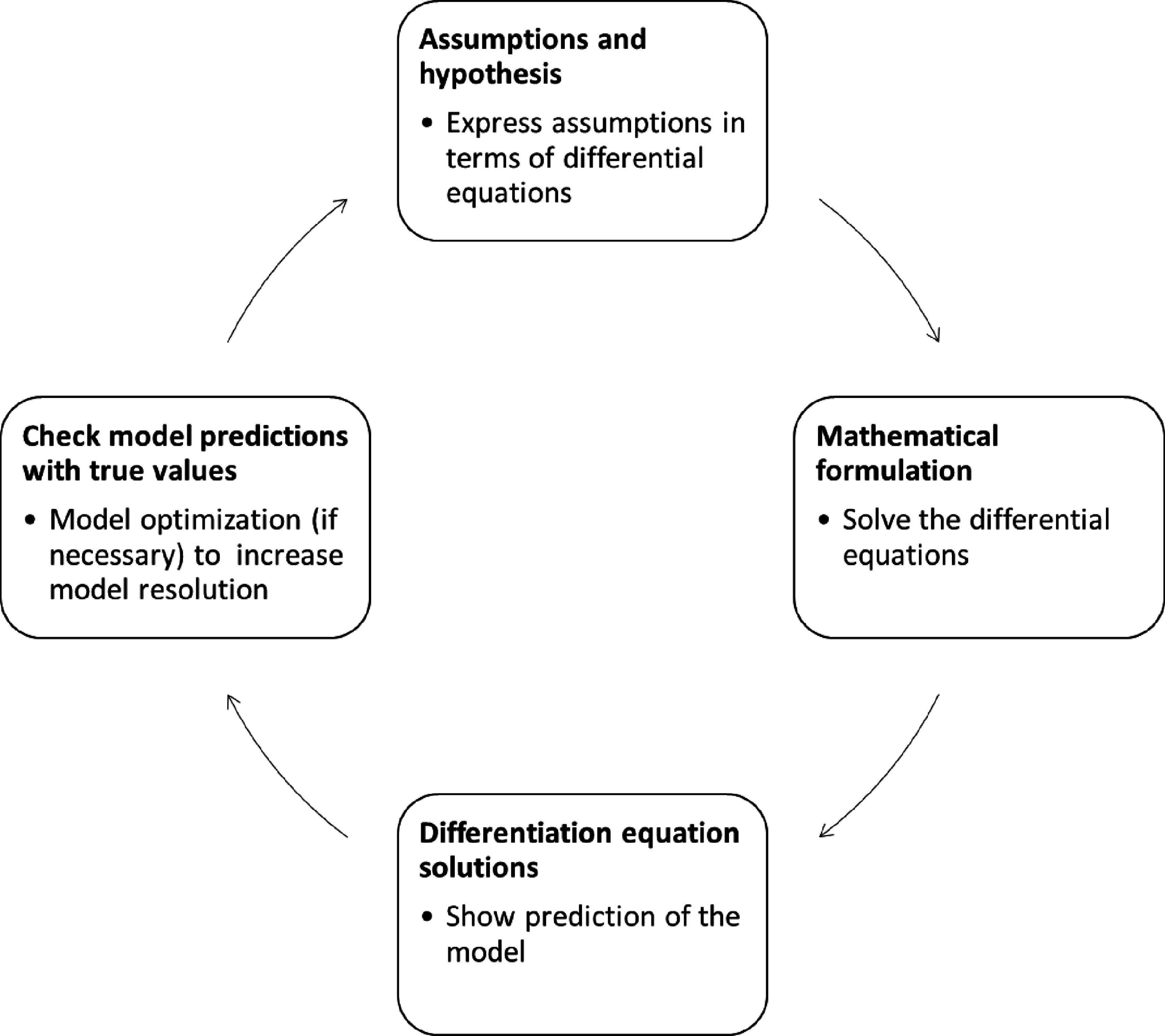
Modelling process with differential equations.

As mentioned prior, the dynamics that pertain to the turnover of specific particles/substances in a biological system are termed kinetics (Anderson 1983). The mathematical models describing these dynamics of biological systems are often formulated as a system of complex ODEs with constant and, in some cases, varying coefficients (Eckerman *et al* 1992). Mathematical forms of ODEs, meaning the unknown function for which a solution is required, depend only on a single independent variable. Thus, choosing the appropriate solving methods and tools influences the accuracy and precision of the solution to the problem and eventually affects calculation performance. Zill (2018) outlined the various steps in figure 3, which depicts the modelling processes with DEs for developing an optimized model.

### 3.2. ODE stiffness

The ODEs mostly encountered can be categorized as either non-stiff or stiff. Non-stiff problems are problems for which all of the components evolve simultaneously on comparable timescales, whereas stiff problems can be defined as follows (Byrne and Hindmarsh 1987, Wanner and Hairer 1996, Omale *et al* 2014):

a. A problem for which no solution component is unstable (no eigenvalue of the Jacobian matrix has a real part which is at all large and positive) and at least some component is very stable (at least one eigenvalue has a real part which is large and negative). The Jacobian matrix is a matrix of first-order partial derivatives of the system’s equations with respect to its variables. The Jacobian matrix provides information about the local dynamics near an equilibrium point—an important concept to improve the stability of solving DEs.
b. A problem for which the solution being sought varies slowly; however, nearby solutions vary rapidly, so the numerical method must take small steps to obtain satisfactory results. For example, for a nearby system component, the component parameter as a constant coefficient-transfer rate may be extremely large compared to the nearby system resulting in rapid variations.
c. A problem for which eigenvalues have negative real parts for a constant coefficient matrix.
d. A problem for which explicit methods do not work or work extremely slowly.

A quantitative measure of stiffness is usually the stiffness ratio—the ratio of the magnitude of the largest to the smallest eigenvalues $\left| {{\lambda _L}} \right|/\left| {{\lambda _S}} \right|$ that should be greater or equal to the ratio of the maximum magnitude to the minimum magnitude of the loss term ${\text{ma}}{{\text{x}}_i}\left| {{A_{ii}}} \right|/{\text{mi}}{{\text{n}}_i}\left| {{A_{ii}}} \right|$in the transfer coefficient matrix (Radhakrishnan and Hindmarsh 1993, Mate-Kole *et al* 2023). As stiff ODEs frequently arise in the study of many problems, including but not limited to chemical kinetics, diffusion process, mathematical biology, mechanics, electrical circuits, control systems, etc, they significantly impact science and engineering (Byrne and Hindmarsh 1987, Nejad 2005, Omale *et al* 2014). Over the last decades, significant progress has been made in developing numerical stiff ODE solvers in ODE solution algorithms and associated linear algebraic methods (Nejad 2005). As a result, a wide range of reliable ODE solvers have been developed.

### 3.3. ODE forms

The subsection aims to briefly emphasize the standard forms of ODEs for completeness. For detailed fundamental mathematical clarity, several textbooks and articles are available in the literature (Tenenbaum and Pollard 1985, Byrne and Hindmarsh 1987, Zill 2018) with working examples of standard DEs for consultation.

The *n*th-order ODE in one dependent variable is of the general form (Zill 2018):

\begin{equation*}F\left( {t,{\text{ }}y,{\text{ }}y^{\prime}, \ldots ,{\text{ }}{y^{\left( n \right)}}} \right) = 0\end{equation*}

where $F$ is a real-valued function of $n + 2$ variables. The normal form of equation (5) can be represented as the differential equation:

\begin{equation*}\frac{{{{\text{d}}^n}y}}{{{\text{d}}{t^n}}} = f\left( {t,{ }y,{ }y{^{^{\prime}}}, \ldots ,{ }{y^{\left( {n - 1} \right)}}} \right)\end{equation*}

where $f$ is a real-valued continuous function and represents the first order differential equation. Canonically, the first order differential equation for initial value problem (IVP) can be illustrated. This is represented as (Byrne and Hindmarsh 1987);

\begin{equation*}{\raise0.7ex\hbox{${{\text{d}}y}$} \!\mathord{\left/ {\vphantom {{{\text{d}}y} {{\text{d}}t}}}\right.} \!\lower0.7ex\hbox{${{\text{d}}t}$}} = f\left( {t,y} \right),{ }{t_o} \unicode{x2A7D} t \unicode{x2A7D} {t_{{\text{final}}}}\end{equation*}

\begin{equation*}y\left( {{t_o}} \right) = {y_o},\end{equation*}

$y = {\left[ {{y^1},{y^2}, \ldots ,{ }{y^{\,N}}} \right]^{\text{T}}}$ is a column *N*-vector of dependent variables, and the superscript T in *y* vector denotes vector transpose, ${\text{d}}/{\text{d}}t$ denotes differentiation of $y$ with respect to *t*, ***f*** is an *N*-vector valued function of $y$ with respect to *t*, ${t_o}$ is the initial value, ${t_{{\text{final}}}}$ is the final value of the interval of integration and ${y_o}$ is the initial value (*N*-vector).

An ODE of the order *n* can be considered linear if it is in the form (Zill 2018):

\begin{equation*}{a_n}\left( x \right){y^{\left( n \right)}} + {a_{n - 1}}\left( x \right){y^{\left( {n - 1} \right)}} + \ldots {a_1}\left( x \right)y{^{^{\prime}}} + {a_o}\left( x \right)y = Q\left( x \right).\end{equation*}

Hence, equation (5) can be said to be linear if $F$ is linear in $y,{ }y{^{^{\prime}}}, \ldots ,{ }{y^{\left( n \right)}}$. A special case where $Q\left( x \right) = 0$ results in a linear homogenous ODE. Nonlinear, on the other hand, is any ordinary equation that is not linear. For example, *F* can be considered nonlinear if it is a function of the product of $y^{\prime}$and $y^{{\prime} {\prime}}$or ${y^{\prime} 2}$—a result of second-order kinetics. Several studies have illustrated, in rigorous detail, the many forms of ODEs (Ince 1956, Wanner and Hairer 1996, Hartman 2002, Zill 2018).

### 3.4. Survey of ODE solving methods

Having introduced the fundamental notation of an ODE, it is worth noting that these ODE forms are customized to tackle real-world problems using well-developed solving algorithms. For ease in solving complex ODE problems, these solving algorithms are then bundled into software tools. With the advancement of ODE-based software, the baseline mathematics underlying the code is no longer readily apparent. With simple guidance, users can input data into the solvers to carry out complex computations. However, understanding these ODE methods is essential, especially when addressing ODEs with unique features that could only be fitted into the existing solvers if they apply salient modifications or solve specific problems. On that note, it is helpful to provide some resources regarding the ODE methods. Several ODE-solving methods have been discussed in detail in the literature (Milne 1970, Byrne and Hindmarsh 1987, Jeffreys *et al* 1988, Butcher 1996, Nejad 2005, Hairer and Wanner 2015) and should be referred to for in-depth mathematical consideration. Specifically, Milne (1970) and Jeffreys *et al* (1988) discussed the general techniques for analytically solving systems of ODEs; however, they also emphasized the importance of leveraging numerical methods for complex systems. According to Bertelli and Lipsztein (1987), an efficient technique for solving linear DEs is an asymptotic analytical method such as the Laplace transform. This method is known to be of great advantage for any time-dependent intake problem such as that encountered in ID. When the Laplace method is used to solve time-dependent intake problems, it was recorded that the form of equations describing the radionuclide accumulation in each compartment *i* in the biokinetic model (compartmental-based model) as a function of time is always the same (see equation (10)) (Bertelli and Lipsztein 1987):

\begin{equation*}{Q_i}\left( t \right) = \mathop \sum \limits_j^n {b_{ij}}{e^{ - {\lambda _j}t}}.{F_j}\end{equation*}

where ${Q_i}\left( t \right) = \mathop \sum _j^n {b_{ij}}{e^{ - {\lambda _j}t}}$ is a single instantaneous intake solution, ${b_{ij}}$ is the coefficient, ${\lambda _j}$ is the eigenvalue and ${F_j}$ is a factor that characterizes the kind of intake of the system. However, for a large number of compartments, Bertelli and Lipsztein (1987) recommended eigenvalue and eigenvector technique as an alternative analytical approach. Thus, for a system of *n* first-order DEs with constant coefficients, a matrix notation can be implemented and then solved by eigenvalue and eigenvector decomposition (equations (11) and (12)),

\begin{equation*}\dot X = A.X\left( t \right)\end{equation*}

\begin{equation*}\left[ {\begin{array}{*{20}{c}} {{x_1}\left( t \right)} \\ \ldots \\ \ldots \\ {{x_n}\left( t \right)} \end{array}} \right] = {\text{ }}\left[ {\begin{array}{*{20}{c}} {{b_{11}}}&amp;{{b_{12}}}&amp; \ldots &amp;{{b_{1n}}} \\ \ldots &amp; \ldots &amp; \ldots &amp; \ldots \\ \ldots &amp; \ldots &amp; \ldots &amp; \ldots \\ {{b_{n1}}}&amp;{{b_{n2}}}&amp; \ldots &amp;{{b_{nn}}} \end{array}} \right]\left[ {\begin{array}{*{20}{c}} {{e^{{\lambda _1}t}}}&amp; \ldots &amp; \ldots &amp;0 \\ \ldots &amp;{{e^{{\lambda _2}t}}}&amp; \ldots &amp; \ldots \\ \ldots &amp; \ldots &amp; \ldots &amp; \ldots \\ 0&amp; \ldots &amp; \ldots &amp;{{e^{{\lambda _n}t}}} \end{array}} \right]\end{equation*}

where ${b_{11}} \ldots \ldots {\text{ }}{b_{nn}}$ are the coefficients of the homogenous solution and ${\lambda _1} \ldots {\lambda _n}$ as the system’s eigenvalues. Despite the method’s robustness, solution difficulties surface in biokinetic model algorithms that utilize the eigenvalue and eigenvector approach where two subsequent compartments have the same rate constant (Killough and Eckerman 1984, Birchall 1986, Bertelli and Lipsztein 1987). For example, let us consider a two-compartmental model with a constant transfer rate of *k*. Equation (13) represents the matrix form of the simple system,

\begin{equation*}\dot x = { }\left[ {\begin{array}{*{20}{c}} { - k}&amp;0 \\ k&amp;{ - k} \end{array}} \right]\left[ {\begin{array}{*{20}{c}} {{x_1}} \\ {{x_2}} \end{array}} \right].\end{equation*}

Now, the characteristic equation can be given as ${\text{det}}\left( {A - \lambda I} \right) = 0$, where $A$ is the matrix of coefficients of the two-compartment system, $\lambda $ is the eigenvalue, and ***I*** is the identity matrix (Hirsch *et al* 2012). Therefore, the characteristic equation results in equation (14),

\begin{equation*}{\text{det}}\left( {A - \lambda I} \right) = { }\left( {\lambda + k} \right)\left( {\lambda + k} \right) = 0.\end{equation*}

Thus, equation (14) results in repeated roots which indicates degenerate eigenvalues. Consequently, the system with degenerate eigenvalues becomes problematic and thus requires additional techniques to study stability. Notwithstanding, Killough and Eckerman (1984), Birchall (1986), Bertelli and Lipsztein (1987) proposed that one or more compartmental rates can be altered by a small fraction (about 5% differences) which does not result in significant error in the solutions obtained.

In many realistic scenarios such as, but not limited to, drug metabolism, and nutrient uptake where transfers are influenced by complex internal and external factors (transfer rates may be time dependent with either a known cumbersome relation or unknown form), ODEs describing these phenomena may not have analytical solutions (Sanchez 2005, Rodriguez-Diaz and Sánchez-León 2014). Also, if analytical solutions exist, it may be very cumbersome to solve analytically. Consequently, numerical methods are employed to find the approximate form of the solution. Butcher (1996), Nejad (2005), and Hairer and Wanner (2015) further articulated the mathematical conception of numerical approximation from the simple Euler method and provided the generalization, approximations, and justifications made over the years for good computational resolution.

In general, the Euler method is one of the simplest numerical methods for solving the first-order IVP. The numerical approximation is well-known to be in the form (Butcher 1996, Zill 2018):

\begin{equation*}{y_{n + 1}} = {y_n} + hf\left( {{t_n},{y_n}} \right)\end{equation*}

where *f* is a function obtained from the differential equation (equation (7)), and *h* is the step size. In some cases, the Euler estimator may overestimate or underestimate the solution value. For the purpose of accuracy, the improved Euler method is mainly implemented to further reduce any error in the general Euler method.

\begin{equation*}{k_1} = f\left( {{t_n},{y_n}} \right)\end{equation*}

\begin{equation*}{k_2} = f\left( {{t_n} + h,{y_n} + h{k_1}} \right)\end{equation*}

\begin{equation*}{y_{n + 1}} = f\left( {{t_n} + h,{y_n} + h(({k_1} + {k_2})/2} \right).\end{equation*}

According to Butcher (1996), the work conducted by Runge, published in 1895, extended the approximation method of Euler, for solving DEs for greater accuracy. A generalization of the basic Euler method is classified as the Runge–Kutta (RK) Method (Zill 2018). The RK method has a wide range of classes but is less often adopted in current ODE software systems for stiff problems (Byrne and Hindmarsh 1987). RK methods belong to a class of one-step numerical integrators for ODEs with intermediate stages in the steps. This method can be categorized as either an explicit or implicit method. Hairer and Wanner (2015) stated that non-stiff problems can be efficiently solved with explicit RK methods, while stiff problems can be solved with certain implicit RK methods. Meaning not all implicit methods are suitable for all types of stiff problems. For illustration purposes, the classical RK method for a typical IVP in equation (7) is given by (Hairer and Wanner 2015):

\begin{equation*}y\left( {{t_o} + h} \right) = {y_o} + \int\limits_{{t_o}}^{{t_o} + h} f\left( {t,y\left( t \right)} \right){\text{d}}t.\end{equation*}

Additionally, Hairer and Wanner (2015) expanded on the mathematical representation of explicit and implicit RK methods and can be consulted for further insight. Over the years, a plurality of other methods and associated families have been developed, including but not limited to multi-derivative methods, Implicit Adams, backward differentiation formulas (BDF), and numerical differentiation formulas (NDF). These methods are known to have significantly contributed to developing advanced ODE solvers (Byrne and Hindmarsh 1987, Postawa *et al* 2020).

## 4. ODE solvers and solving methods

### 4.1. Conventional ODE solvers

This section focuses on a survey of several standard ODE solvers across programming languages. This is foundational to understanding and exploring the extent to which these solvers have evolved and their capabilities.

#### 4.1.1. The GEAR flavour

According to Byrne and Hindmarsh (1987), GEAR pioneered a software package called DIFSUB in 1968, based on the BDF method. This package was notably identified as the first routine base ODE solver, which has since been widely used for all stiff IVPs (Nejad 2005). Subsequent revisions were conducted after encountering computational difficulties for some kinetic models with DIFSUB (Byrne and Hindmarsh 1987). The revised software named STIFF later contributed to the development of GEAR as an ODE package. Several varieties of the GEAR package were further developed due to the different nature of IVPs encountered, such as problems with sparse or dense Jacobian matrices and, as a result, a large number of variants are available for use (Byrne and Hindmarsh 1987, Nejad 2005). Sparse matrices are mostly with zero entries, while dense matrices are matrices with mostly non-zero entries. Exploiting the sparsity of Jacobian matrices improves the computational efficiency of the numerical solvers. In some cases, a sparse matrix can be classified as banded, where the non-zero entries are concentrated along the main diagonal and a few adjacent diagonals. Specialized solvers with lower computational complexities are used to exploit the band structure of such matrices for faster solutions. GEARB was designed with a GEAR flavour for banded Jacobian matrices (Nejad 2005).

#### 4.1.2. CVODE & PVODE

Furthermore, as computational demands increased, complex physics model problems could be divided into small fractions, which could be solved simultaneously, stimulating the evolution of parallel computing. Most of these physics model problems were solved as a system of ODEs; thus, the ODE solvers required adaptability for parallelism. As a result, PVODE was developed as a general-purpose ODE solver for parallel computers, which uses a message-passing interface (MPI) and a revised version of the vector module in CVODE to achieve parallelism (Byrne and Hindmarsh 1999).

#### 4.1.3. ODEPACK collection

Due to the large number of ODE solvers developed by Hindmarsh and collaborators at the Lawrence Livermore National Laboratory (LLNL), concerns were raised by users and suppliers desiring standardization (Hindmarsh 1983). A collection of families of ODE solvers was then developed and named ODEPACK. Table 2 outlines some general-purpose ODE solvers available in the ODEPACK collection.

**Table 2.** General purpose solving packages for solving system of ODEs (Nejad 2005).

Solver	Features
GEAR (1974) GEARB GEARS	Supersedes DIFSUB—Gear 1968 Banded Jacobian Sparse Jacobian
LSODE (1982) LSODES	Basic solver of the ODEPACK collection and combines the capabilities of GEAR and GEARB. Sparse Jacobian for stiff cases: treats the Jacobian matrix as a general sparse matrix.
LSODPK	Implement preconditioned Krylov iteration methods for linear systems—For a linear system like $b = A{\text{ }}x$, Krylov iterative method (Hindmarsh and Petzold 1995) assumes some initial approximation ${x_0}$ and its residual ${r_0}=b - A{x_0}$. Using these starting assumptions, the exact solution is computed iteratively.
VODE (1989)	Variable-coefficient and fixed leading-coefficient form of BDF for stiff systems. Supersedes EPISODE and EPISODEB—EPISODE is an ODE solver that uses implicit multistep method designed for dense Jacobian matrices and EPISODEB for banded matrices.
VODPK (1992)	Implement preconditioned Krylov iteration methods for linear systems. Combination of VODE solver and Krylov methods
CVODE	Standard C: VODE and VODPK options written in C
PVODE (1995)	Parallel VODE in ANSI standard C with preconditioned Krylov iteration methods.

With a few exceptions, the ODEPACK solvers comprised standard FORTRAN 77 with minimal machine dependencies (Hindmarsh 1983). Each ODEPACK solver came in a version of either single or double precision. From Hindmarch (1983), numerous upgrades of the ODEPACK solvers were performed to improve the quality, clarity, and efficiency of the solving methods. These were: renaming of routines and common blocks to distinguish double and single precision versions; the use of generic intrinsic function names; elimination of the block data subprogram; use of a portable routine to set the unit roundoff; reformatting comments; and passing of quoted strings to the error message handler.

#### 4.1.4. BzzOde

ODE solver performance relies heavily on efficiency and robustness. To enhance performance, a class of C++ ODE solvers for stiff and non-stiff ODE systems was developed (Ferraris and Manca 1998) called BzzOde. C++ was chosen as a platform for BzzOde to increase implementation efficiency and ease of use. BzzOde was designed to solve stiff and non-stiff problems. The study aimed to solve stiff problems, which were identified as the most challenging and frequently encountered issues in chemical kinetics. According to Ferraris and Manca (1998), VODE and BzzOde have a significant advantage over LSODE and DASPK; however, BzzOde is said to follow a different criterion with respect to VODE in determining when to update the Jacobian matrix. Thus, BzzOde checks whether the stored Jacobian matrix is out of date, where the Jacobian matrix is kept constant for a maximum of 50 steps, enhancing performance. The study (Ferraris and Manca 1998) concluded that BzzOde’s performance is better than the standard FORTRAN ODE solver. BzzOde’s ease of use was achieved through a globally revised object-oriented approach in C++.

#### 4.1.5. SUNDIALS

SUNDIALS, which is the SUite of Nonlinear and Differential/Algebraic equation solvers, consists of CVODE (ANSI Standard C of the VODE and VODPK combined solvers), KINSOL, and IDA (Hindmarsh *et al* 2005). According to Hindmarsh *et al* (2005), the time integrators and nonlinear solvers within SUNDIALS have been developed to take advantage of the long history of research and development of such codes at LLNL by featuring state-of-the-art technology for BDF time integration, as well as for inexact Newton–Krylov methods (Brown and Saad 1990). Moreover, the paper by Hindmarsh *et al* (2005) outlined several underway updates, such as solvers with sensitivity analysis capabilities.

### 4.2. Historical studies comparing ODE solvers

The discussions earlier explicitly showed the extent to which ODE solvers have evolved, as well as some strengths and weaknesses. Therefore, carefully selecting ODE-solving methods is crucial to creating a robust and efficient toolkit (ODE solver) for research and industrial use. A detailed study compared ODE solvers for biochemical processes (Postawa *et al* 2020). As different programming environments offer a wide selection of ODE solvers, the study by Postawa *et al* (2020) tested a wide range of algorithms, starting from simple, single-step explicit methods and ending with implicit multi-step techniques. The programming environments chosen for their work were matrix laboratory (MATLAB), Python, C++, and C#, with the list of solvers in table 3. According to Postawa *et al* (2020), most of the solving methods studied resulted in correct and consistent results; however, GearBDF was unable to cope with the system of ODEs, resulting in some negative solutions. Therefore, a preference for the use of implicit solution methods for stiff biological problems was confirmed, whereby three ODE solvers stood apart. LSODA was identified as satisfactory for solving simple biological systems as a handy open-source solver. However, LSODA struggles to cope with very complex problems, as it requires more time steps to compute an accurate solution.

**Table 3.** Programming environment with selected ODE solvers (Postawa *et al* 2020).

Problem type	Method type	Solver name	Environment
Explicit	Adams–Bashforth–Moulon	ode113	MATLAB
	Runge–Kutta	ode23	MATLAB
	Runge–Kutta	ode45	MATLAB
	Runge–Kutta	RK547M	C#
	Runge–Kutta	dopri5	Python
	Runge–Kutta	runge_kutta_dopri5	C++
	Runge–Kutta	dop853	Python
	Bulirsch–Stoer	bulirsch_stoer	C++
Implicit	Backward differentiation formulas	GearBDF	C#
	Backward differentiation formulas for stiff problems	vode_bdf	Python
	Numerical differentiation formulas	ode15s	MATLAB
	Adams	vode_adams	Python
	Adams/BDF	Lsoda	
	Rosenbrock	ode23s	MATLAB

Ode15s was recommended for higher-order complex systems as it requires fewer steps to produce solutions. Moreover, Ode23s was recommended if accuracy is required.

As many studies consolidated ODE solvers across programming platforms, selecting solvers specific to a programming environment and application scope became relevant. A mathematical analysis of ODE’s stiff and non-stiff IVPs using MATLAB was conducted (Omale *et al* 2014). MATLAB is a high-level language and interactive computer environment developed by MathWorks for scientists and engineers to analyse and design systems. According to Omale *et al* (2014), MATLAB’s tools and built-in math functions enable the exploration of multiple approaches and reach a solution faster than with spreadsheets or traditional programming languages, such as C/C++ or Java. In the study of Omale *et al* (2014), several ODE solvers in MATLAB were studied by subjecting them to six IVPs (three of which were non-stiff problems and the other three were stiff problems), for which the solvers tested are summarized in table 4. The methods of the MATLAB solvers are not covered in this section because most are derived from the methods outlined in previous sections.

**Table 4.** MATLAB ODE solvers.

Solver	Problem	Algorithm
Ode45	Non-stiff differential equations	Dormand–Prince pair: Runge–Kutta
Ode23	Non-stiff differential equations	Bogacki–Shampine: Runge–Kutta
Ode113	Non-stiff differential equations	Adams–Bashforth–Moulton predictor-corrector
Ode15s	Stiff differential equations	Numerical differentiation formulas along with Gear’s method
Ode23s	Stiff differential equations	Rosenbrock
Ode23t	Moderate stiff problems	Trapezoidal Rule with free interpolant
Ode23tb	Stiff differential equations	Trapezoidal—backward differentiation formulas
Ode15i	Fully implicit differential equations	Backward differentiation formulas (BDFs)

Although Ode23 and Ode113 failed when explicitly tested against the predator-prey (Lotka-Volterra) model, a pair of first-order nonlinear DEs, the study (Omale *et al* 2014) demonstrated the effectiveness of MATLAB ODE solvers for solving IVPs. Moreover, the study recommended that further studies on the optional parameters (such as Jacobian Matrix, error control parameters, etc) of the various solvers are required to enhance performance and perform specialized computation. Further analysis of the six sets of IVPs is detailed in Omale *et al* (2014). While several methods to numerically solve ODEs and differential–algebraic equations have been examined, most of these ODE solvers are available in different programming languages.

**Figure 4.**
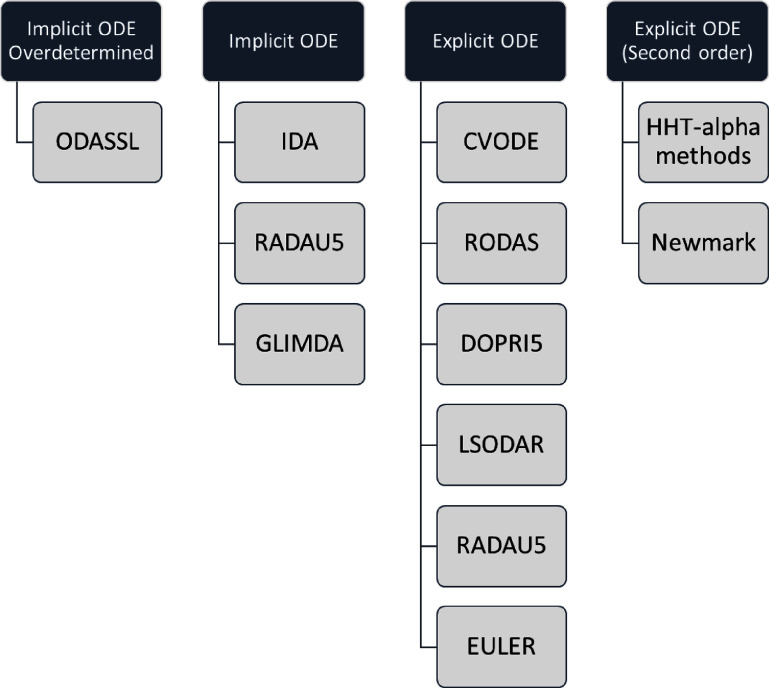
Integration of problems with respective solvers in Assimulo.

Therefore, unified interfacing was deemed useful for the research and industrial sectors (Andersson *et al* 2015). A study conducted by Andersson *et al* (2015) resulted in the development of a unified high-level interface to solvers of ODEs, as well as addressing the requirements for solving industrial models with discontinuities and data handling. Their interface, which is coded in Python/Cython, combines original classical and modern solvers independent of their programming language. Python is an object-oriented interpreted programming language where an interpreter is needed to convert Python codes into machine codes. This programming language has gained significant momentum in scientific computing (Oliphant 2007). Cython, on the other hand, is a superset of Python, a compiler programming language designed to give C-like performance with code written primarily in Python with optional additional C-inspired syntax. Assimulo has been formulated as an interface for integrating several problems with specified solvers, as illustrated in figure 4 (Andersson *et al* 2015).

Andersson *et al* (2015) further demonstrated the implementation of the core of Assimulo, for which each solver is organized into specific class structures for both implicit and explicit ODE problems in Python/Cython. Most of these solvers are connected with external codes, which are compiled either from FORTRAN or C. Following a detailed study of different problem classes with respective ODE solvers, Andersson *et al* (2015) proposed to increase the variety of original codes and make them available through the framework provided. Furthermore, a dedicated study on multiphysics pharmacokinetic models demonstrated the need for ODE solvers in compartmental modelling (Glass *et al* 2022). The motivation for this recent study was that physiologically-based pharmacokinetic (PBPK) models use an empirically derived framework that cannot be universally applied to varying nanoparticle constructs and experimental settings. Thus, the study was designed to develop a physics- based multiscale PBPK compartmental model to determine the continuous biodistribution of nanoparticles.

According to Glass *et al* (2022), two versions of physics-based compartmental models were developed, for which the stiff ODE solving methods used were from MATLAB and Julia (Rackauckas 2017, Bezanson *et al* 2017) and validated against experimental data. Julia was developed as an alternative to Python and MATLAB. For a precise evaluation of the handling of ODE stiffness for both models, Glass *et al* (2022) used one stiff MATLAB solver known as Ode15s and five other stiff solvers—such as QNDF, Rodas4, KenCarp4, TRBDF2, and RadauIIA5 from the *DifferentialEquations.jl* package in Julia. Ode15s from MATLAB was used for solving the system of large and stiff ODEs; however, this resulted in biodistribution solutions for a time interval of 0–1 ms.

According to Glass *et al* (2022), this is due to the nature of the times (small) required for stability in the solver, and thus MATLAB becomes unresponsive if the time steps are increased beyond 1 ns. Moreover, the systems were solved successfully using the stiff packages in Julia for large time points. In that regard, the study aimed not to compare ODE solvers in MATLAB and Julia but to use Julia where MATLAB fails to produce results. A key takeaway note in this study was the demonstration that a neural network could learn to solve a system of ODEs when the system can be made non-stiff (Glass *et al* 2022).

A study by Mate-Kole *et al* (2023) compared Python-based differential equation solvers and methods. In addition to emphasizing the compartmental-based approach for biokinetic modelling, Mate-Kole *et al* (2023) mainly exploited the capabilities of SciPy explicit and implicit ODE solvers and a Python-based matrix exponential method for evaluating the ODE systems corresponding to selected biokinetic models. This study (Mate-Kole *et al* 2023) reaffirmed the general solution approach to biokinetic problems and demonstrated using Python that implicit and algebraic solving methods are well-suited for the complex systems of ODEs constituting biokinetic models.

Besides demonstrating the solving capabilities of SciPy ODE solvers (stiff and non-stiff problems), there has been interest in improving the performance. One study (Hagen and Mayorov 2019) emphasized the need to investigate if cythonizing (a superset of Python programming language with a C-inspired syntax) the Python classes improves the performance of the new solvers without compromising effective solving capabilities. In general, Python, as an interpreted and dynamic programming language, offers substantial flexibility and supports an agile development process (Schmitt *et al* 2022). However, this may imply reduced speed and higher memory consumption during run-time, which could cost some computational execution. According to Schmitt *et al* (2022), to increase execution speed, most equations or algebraic computer systems are designed in compiled programming languages.

Another study (Schmitt *et al* 2022) described a new Python package named sympy2c. The package sympy2c was designed to bridge the gap between symbolic development and the numerical implementation of a theoretical model. Thus, the study addressed translating symbolic equations implemented within the Python CAS SymPy to a fast C/C++ code that can be used from Python as an extension module (Schmitt *et al* 2022). In a new package, developers of sympy2c paid critical attention to some shortfalls regarding existing ODE solvers by considering sparsity in the Jacobian matrix and implementing routines for numerical integration and spline interpolation. Additionally, LSODA was enhanced in sympy2c for efficient step-size control and for effective stiffness detection and control. According to the study (Schmitt *et al* 2022), the overhead of code generation and compilation time limits the application scope of the ODE solver to situations where the same ODE has to be solved many times with varying coefficients or initial conditions. In order to improve efficiency, the developers intend to create smaller files that will support the optimization process of the compiler. This will allow for parallel compilation of source codes.

## 5. Solving methods for modelling the distribution and dosimetry of internal emitters

Compartmental analysis is a widely adopted methodology in the realm of ID and various other scientific disciplines. This approach entails the discretization of the system into a finite number of components, called compartments allowing them to interact by means of exchanging species such as radioactive materials, chemical substances, and body fluids (Sanchez 2005). For instance, the systemic biokinetic model, as delineated in ICRP Publication 141 (ICRP 2019), expounds on how an actinide element like americium is absorbed into the bloodstream. This publication is among a series of reports on occupational intake of radionuclides, with further elaboration on the actinide compartment model available in ICRP Publication 141 (ICRP 2019).

The estimation of radionuclide content in the human body is achieved through the utilization of a system of DEs constituting the biokinetic model. The process can be performed through analytical or numerical computational methods, detailed in the ODE solvers and methods section. Several solvers/methods exist for solving the biokinetic problem and are embedded in various internal dose computer programs, as well as other commercially available general-purpose modelling toolkits. Table 1 outlines an inventory of internal dose codes alongside their corresponding ODE solvers/methods; the codes tabulated here represent only those codes with identified and documented ODE/solving methods implemented. Further historical background and methods employed of/by selected codes are presented in the forthcoming discussion. It should be further noted that the codes tabulated or discussed herein do not represent any explicit recommendation by the authors.

### 5.1. TIMED (1976)

Once a radionuclide is deposited in the human body, the cumulative activity in an organ can be estimated by integrating the retention from an initial time time (*t=0*) to the desired time post-deposition. However, in some instances, the transfer of radionuclides between organs/tissues can be complex. This may include recycling, the presence of radionuclide’s progeny and subsequent chain radionuclides. To address these difficulties, a computer program known as TIMED was developed (Watson *et al* 1976). TIMED was designed to estimate the cumulative activity of radionuclides in the body with program routines written in FORTRAN IV for either the IBM System/360 or IBM System/370 and Assembler language for the IBM System/360. TIMED is designed to account for the delay of transfer of activity between compartments in the model and generation of radioactive progeny. According to Watson *et al* (1976), the solutions of the ODEs are estimated using a FORTRAN subroutine—the GEAR package which is known for its ability to solve stiff ODE problems. The solution method implemented utilized an implicit linear multistep type categorized as the implicit Adams method (maximum order of 12), and the BDF method (maximum order of 5) (Watson *et al* 1976). Watson *et al* (1976) noted that TIMED was designed to be executable on the IBM System/360 or System/370 machines, and, hence, had the limited accessibility.

### 5.2. DIFSOL (1984)

Several studies have investigated approaches for solving complex biokinetic systems. In a study by Vicini *et al* (2008), the origin of mathematical modelling methods with specific attention to radiotracers applications is highlighted. This study describes compartmental models of increasing detail from the simplest possible model (Oddie 1949) to the most complex. A prior study by Killough and Eckerman (1984) prompted the development of a conversational code, called DIFSOL, for evaluating the solution of metabolic models specific to health physics. This program was written in FORTRAN IV programming language and translated into BASIC for the Radio Shack TRS-80 Model I/111 microcomputers. According to Killough and Eckerman (1984), DIFSOL solves an IVP in the form:

\begin{equation*}\frac{{{\text{d}}Z}}{{{\text{d}}t}} = AZ\end{equation*}

\begin{equation*}Z\left( 0 \right) = {Z^0}\end{equation*}

where is a vector of *N* functions; $A$ is a constant $N \times N$matrix coefficient; and ${Z^{\,0}}$ is a vector of initial values of $Z$.

The analytical approach employed in the study utilized matrix eigensystem techniques to express the solution vector $Z\left( t \right)$ in terms of exponential functions of the form: ${e^{at}}$, ${e^{at}}\cos bt$, and ${e^{at}}\sin bt$. The solving solution method of DIFSOL with example applications are detailed in the study by Killough and Eckerman (1984). However, the assumption that the eigenvectors form a linearly independent set was violated in certain cases, leading to program failure. To address this issue, a proposed solution involved introducing a small perturbation in the model parameters, ensuring that the perturbed system possessed linearly independent eigenvectors and limited second-order error in its solution.

Consequently, DIFSOL was proven to be practical for small systems of less than 12 parameters. Using this code outside these parameters resulted in meaningless solutions (Killough and Eckerman 1984). Five years later, Birchall and James (1989) presented an algorithm for solving first-order compartmental models involving recycling on a microcomputer. This algorithm approached solving the system analytically by employing matrix algebra, which was evaluated by finding the exponential of the matrix of constant coefficients. This is expressed as:

\begin{equation*}{x_i}\left( t \right) = {e^{\left[ A \right]t}}.{x_i}\left( 0 \right)\end{equation*}

where ${e^{\left[ A \right]}}$ is the exponential of the matrix [A]. Several numerical methods and approximations were investigated to evaluate ${e^{\left[ A \right]t}}$. However, Birchall and James identified that most methods required an intricate computation of the eigenvalues and eigenvectors of the system, rendering them ill-suited for these systems. Furthermore, the utilization of characteristic equations as a resolution had proven problematic to implement and computationally burdensome. While Birchall and James employed a series expansion method, the consequence of implementing this approach resulted in difficulty in evaluating ${e^{\left[ A \right]t}}$ for large t values. Hence, an optimized approach was implemented as:

\begin{equation*}{e^{\left[ A \right]}} = {\left[ {{e^{{\raise0.7ex\hbox{${\left[ A \right]}$} \!\mathord{\left/ {\vphantom {{\left[ A \right]} x}}\right.} \!\lower0.7ex\hbox{$x$}}}}} \right]^x}.\end{equation*}

For $x \ne 0$ and letting $x = {2^n}$, for n as an integer, ${e^{\left[ A \right]}}$ was evaluated as:

\begin{equation*}{e^{\left[ A \right]}} = {\left[ {{e^{{\raise0.7ex\hbox{${\left[ A \right]}$} \!\mathord{\left/ {\vphantom {{\left[ A \right]} {{2^n}}}}\right.} \!\lower0.7ex\hbox{${{2^n}}$}}}}} \right]^{{2^n}}}.\end{equation*}

as an improved series expansion methodology. Birchall and James further compared the performance of the series expansion of ${e^{\left[ A \right]}}$to the modified expansion as a function of time, *t*. The standard series expansion proved ineffective at larger time points, while the modified series expansion proved to be a suitable option when considering larger time points.

### 5.3. Integrated modules for bioassay analysis (IMBA) (1998)

Several computer codes such as GENMOD (Dunford and Johnson 1987), INDOS (French *et al* 1988, Silverman 2002), REMEDY (Rich 1990), and CINDY (Strenge *et al* 1990) became commercially available in the mid-1980s for evaluation of bioassay data and internal dose estimation.

These codes were based on methodologies of the ICRP Publications 26 and 30 series reports (ICRP 1977, 1979), and thus these computer codes were unable to use or upgrade to new and complex models like the ICRP Publication 66 Human Respiratory Tract Model (ICRP 1994), associated systemic models updated by that time (Birchall *et al* 1998). This motivated the development of IMBA to implement new models (Birchall *et al* 1998, 2005). The IMBA code is a software module suite that implements the ICRP biokinetic, dosimetric, and bioassay models (including the NCRP wound models) to estimate intakes and doses on a Visual Basic platform compatible with Windows OS (Birchall *et al* 2005).

While mathematical algorithms were not explicitly detailed in the IMBA documentation, it was indicated that the matrix exponential algorithm, an algorithm described by Birchall and James (1989), was utilized to address the system of ODEs presented by the biokinetic models enabling the estimation of material retention in organs. Subsequently, a sequence of exponentials was fitted to the contents in the compartments to achieve retention functions. In accordance with the guidance on IMBA usage (U.S. Department of Energy 2006) provided by the U.S. Department of Energy, a significant design limitation was identified with regard to the improper evaluation of the system of ODEs of the biokinetic models in scenarios where there are identical rate constants in a particular series of compartments, for which a workaround was implemented to address this constraint. However, it is essential to note that the algorithm incorporated in IMBA has a notable drawback involving difficulties associated with modifying already implemented biokinetic models or introducing new models (U.S. Department of Energy 2006). The IMBA tool has undergone several quality assurance processes for which further sponsored project led to the development of a user-friendly interface module known as IMBA Expert^TM^ (Birchall *et al* 2007). According to Birchall *et al* (2007), the interface was improved into a general ‘off-the-shelf’ module (IMBA Professional) which had its new version named IMBA Professional Plus. IMBA Professional Plus is reported to be faster than its predecessors with the ability to conduct Bayesian analysis (Birchall *et al* 2007).

### 5.4. GENMOD (1998)

In 1998, the developers of the GENMOD ID code resolved the issue of rigidity by introducing an enhanced version that facilitated the integration of the new ICRP respiratory tract model into previous codes (ICRP 1994, Richardson and Dunford 1998). GENMOD was designed to calculate the retention, excretion, and integrated retention for radionuclides of interest under a variety of exposure conditions. According to Richardson and Dunford (1998), GENMOD utilizes CVODE (Cohen and Hindmarsh 1994) as a numerical solver for the ODE, which was compared to a symbolic analytical method (algebraic) in Mathematica with an absolute precision agreement of 10^−8^ or better. CVODE is a C-based ODE solver for stiff and non-stiff problems which combines the capabilities of two FORTRAN-based solvers (VODE and its variant VODPK) (Cohen and Hindmarsh 1994). It is important to state that the first version of GENMOD used as a dosimetry code was reported to be developed in 1979 and utilized FORSIM as a solver (Dunford and Johnson 1987). In light of the implementation of the ODEPACK solver package at ORNL, a decision was made to update GENMOD from using the FORSIM solver to ODEPACK (Dunford and Johnson 1987). As described by Dunford and Johnson in 1987, this update not only facilitated the inclusion of new models but also aimed to improve the overall coding efficiency, clarity, and documentation, benefiting from the faster and more user-friendly features of ODEPACK. Furthermore, with the presentation of new recommendations incorporated in the ICRP Publication 60 and 66 and the memory constraints of MS-DOS systems, the developers made significant efforts to translate the program into C++ programming language and as a result, had a Windows-based GENMOD (Richardson and Dunford 1998).

### 5.5. Simulation, analysis, and modelling software for tracer and pharmacokinetic studies (SAAM II) (1998)

The scientific community has taken an interest in how kinetic analysis and integrated system modelling impact the experimental design of drug delivery in humans and animals. To meet this need, Barrett *et al* (1998) developed a software tool called SAAM II. This tool enables researchers to create linear or non–linear models, design and simulate experiments, and analyse data efficiently. SAAM II also has a graphical ‘drag-and-drop’ method for constructing compartmental models. Users can specify models directly by entering the governing algebraic equations or choosing from predefined numerical methods. The numerical methods of SAAM II are based on three computational integration techniques, each with its specific strengths and weaknesses, such as the Rosenbrock integrator, which makes use of the semi-implicit method, the standard forward-integration RK method mostly for non-stiff problems, and the Padé integrator based on the Padé approximation of the matrix exponential—only applicable in SAAM II when the model has constant rates, bolus or constant-infusion inputs. SAAM II also implements other statistical methods, including the objective function (Kamp *et al* 2023). This is an extended least-squares maximum likelihood function that optimizes the parameters and variance of the data with respect to the available information (Sanchez 2005, Kamp *et al* 2023).

### 5.6. INDOSE (2002)

In addition to existing ID codes, InDose was developed with the main purpose of estimating activity retained in the tissues and excretion for a given intake (Silverman 2002). As of 2002, Silverman (2002) stated that, although the main task of the code is to compute the activity retention and excretion in/out of the body, the code would have the capability to perform optimizations for automatic estimates of intake and computation of the dose from the predicted intake. The computer code is documented to be written in FORTRAN 90 and employs LSODES as a stiff differential equation solver for the biokinetic models implemented (Silverman 2002). According to Silverman (2002), the version of LSODES used is specifically adapted to sparse matrices.

### 5.7. Monitoring to dose calculation (MONDAL) (2004)

In conjunction with the efforts to develop a more robust computer program to adapt the new models, the National Institute of Radiological Sciences in Japan also developed a personal-computer-based software called MONDAL with attention to a non-specialist user (Ansoborlo *et al* 2003, Ishigure *et al* 2004). MONDAL implemented the ICRP Publication 66 models, the biokinetic models in the ICRP Publications 30, 56, 67, 69, and 71, and the gastrointestinal tract model in the ICRP Publication 30 (Ishigure *et al* 2004). To solve the system of equations corresponding to these biokinetic models, MONDAL utilized the numerical RK method, as detailed by Ishigure *et al* in 2004.

### 5.8. Organ level internal dose assessment/exponential modelling (OLINDA/EXM) (2005)

OLINDA version 1.0 is commercial software designed for internal dose assessment in nuclear medicine (Stabin *et al* 2005, Li 2018). This code was designed for use on a personal computer and coded entirely in Java, including a module for EXM. OLINDA/EXM was rewritten from a BASIC-based internal dose code, known as MIRDOSE, due to challenges associated with migration onto a new operating system. This software was intended to be useful for calculating doses for clinical trials involving radiopharmaceuticals and making theoretical calculations for existing pharmaceuticals. According to Stabin *et al* (2005), the EXM capability of OLINDA/EXM allows for fitting kinetics data using the least-square method projected using the sum of exponentials. The integral of the sum of exponentials results in the number of radionuclide disintegrations in a designated source organ in the body (Stabin *et al* 2005).

### 5.9. BIOKMOD (2005)

Compartmental models become complex due to the presence of multiple exchange pathways. To handle this complexity, models need to be decomposed into matrices that account for both gain and loss terms. In the development of the BIOKMOD code in Mathematica, this approach was implemented by introducing a specific function named *CompartMatrix*. This function is explicitly designed to generate a matrix of coefficients for compartmental systems with *n* compartments. In some cases, the matrix function *CoefMatrix* is used instead of the constant coefficients between compartments, primarily when coefficients are associated with measured physiological parameters or functions, resulting in a physiological model instead of a standard compartmental model (Sanchez 2005). A compartment can be represented as:

\begin{equation*}\dot x\left( t \right) = A.x\left( t \right) + b\left( t \right){\text{ }}t \unicode{x2A7E} 0\end{equation*}

\begin{equation*}x\left( t \right) = {x_o}\end{equation*}

where ${x_o}$ is a vector initial condition as defined in equation (21) and $b$ is an input into the associated compartments, which could be either constant or variable dependent. BIOKMOD solves the biokinetic system analytically by using the function *SystemDSolve*. According to the developer, this Mathematica function has the flexibility to either use the default evaluation method given in equation (27) or equation (28) or specify the computational method from built-in Mathematica functions like *MatrixExp* or *Laplacetransform* given in equations (29) and (30). Equation (29) represents the Inverse Laplace transform,

\begin{equation*}x\left( t \right) = {x_o}{e^{At}} + \int\limits_0^t {e^{A\left( {t - \tau } \right)}}b\left( \tau \right){\text{d}}\tau \end{equation*}

or equation (28) for constant ***b***

\begin{equation*}x\left( t \right) = {x_o}{e^{At}} + b\mathop {\mathop \smallint \nolimits }\limits_t^0 {e^{A\tau }}{\text{d}}\tau \end{equation*}

\begin{equation*}{\boldsymbol{x}}\left( t \right) = {\mathcal{L}^{ - 1}}\left( {{{\left( {s{\boldsymbol{I}} - {\boldsymbol{A}}} \right)}^{ - 1}}{\boldsymbol{x}_o}} \right) + {\mathcal{L}^{ - 1}}\left( {{{\left( {s{\boldsymbol{I}} - {\boldsymbol{A}}} \right)}^{ - 1}}{\boldsymbol{B}}\left( s \right)} \right)\end{equation*}

\begin{equation*}{\boldsymbol{X}}\left( s \right) = {\left( { - \boldsymbol{A}} \right)^{ - 1}}{\boldsymbol{x}_o} + {\left( {s{\boldsymbol{I}} - {\boldsymbol{A}}} \right)^{ - 1}}{\boldsymbol{B}}\left( s \right)\end{equation*}

where ${\boldsymbol{X}}\left( s \right)$ is the Laplace Transform of equation (25).

The functionality of BIOKMOD has been extended to incorporate bioassay data, where the intake is estimated from bioassay measurements by performing maximum likelihood estimation. The goodness of fit for a bioassay data fitting is evaluated using a chi-square test and *p*-value calculation (Sanchez 2005, Moraleda *et al* 2020). Prior to the availability of the development of the Mathematica toolkit, Polig (2001) expounded on the use of matrix methods for modelling the distribution and dosimetry of internal emitters for single intake and more complex intake scenarios, such as chronic and exponential intake. Despite the limitations of linear algebraic methods like matrix methods, Polig underscored the value of these methods in internal dose estimation, emphasizing their suitability for biokinetic and dosimetry models regardless of complexity.

### 5.10. Dose and risk calculation (DCAL) (2006)

Age-dependent dose coefficients were developed utilizing biokinetic models from the ICRP, where the system is solved using transfer coefficients that vary with age. Eckerman *et al* (1992) proposed a straightforward approach for solving compartmental models with time-dependent coefficients. This method was an extension of an earlier technique implemented in the INREM-II dosimetry code to compute the committed dose equivalent to a reference adult from an intake of the radionuclide. The INREM-II internal dose code utilizes a linear combination of decaying exponentials for solving the DEs, part of which is solved in (1) a closed form and (2) using a discrete approximation for some instances with continuous transfer of activity (Killough *et al* 1978). In comparison with INREM-II, the AGEDOS code uses similar features for solving the DEs of compartmental models specifically for organ dose rate as a function of age following internally incorporated radionuclides (Leggett *et al* 1984).

The proposed approach had the advantage of not restricting the number of compartments comprising the problem space. Eckerman *et al* (1992) considered first-order kinetics in an isolated compartment subject to a constant inflow of substances at a rate of *P* and a constant clearance coefficient of *R*. By assuming an initial value of ${Y_o}$, the retention at later time point *T* was expressed as:

\begin{equation*}Y = \frac{P}{R}\left( {1 - {e^{ - RT}}} \right) + {Y_o}{e^{ - RT}}.\end{equation*}

For which the integrated retention from 0 to *T* was also expressed as:

\begin{equation*}YW = \left( {{Y_o} - \frac{P}{R}} \right)\frac{{1 - {e^{ - RT}}}}{R} + \frac{P}{R}T.\end{equation*}

While the relations in equations (31) and (32) applied to single compartments (isolated), their applicability to multicompartmental models was demonstrated to be feasible using an iterative approach to solve the model to the desired degree of accuracy. By employing the first-order kinetics solution approach from Eckerman *et al* (1992) in an expanded form, the DCAL was developed (Eckerman *et al* 2006).

### 5.11. PLEIADES (2007)

A detailed method implemented in the ID code PLEIADES was adopted for solving the biokinetic model problem for eventual use in dose coefficient generation by the ICRP (Fell *et al* 2007). This method distinguished between shared kinetics, where progeny were assumed to share the parent’s biokinetic model, and independent kinetics, where progeny were assumed to follow their own element-specific biokinetic model independently. This method further emphasized the employment of the matrix form for a coupled system of ODEs with the adopted solution method similar to that of equation (22) (Fell *et al* 2007). For shared kinetics, Fell *et al* (2007) demonstrated how separating the biokinetic and radiological processes into different square matrices ***B*** and ***R*** results in a rectangular matrix ***Q*** to represent the activity distribution compared to the standard vector formulation in equation (20). This rectangular matrix is given as:

\begin{equation*}\frac{{{\text{d}}{\boldsymbol{Q}}}}{{{\text{d}}t}} = {\boldsymbol{BQ}} + {\boldsymbol{QR}}.\end{equation*}

With the solution:

\begin{equation*}{\boldsymbol{Q}}\left( t \right) = {e^{{\boldsymbol{B}}t}}{\boldsymbol{Q}}\left( {\text{0}} \right){e^{{\boldsymbol{R}}t}}.\end{equation*}

According to Fell *et al* (2007), this factorization accelerates the calculations for long chains, and this result contradicts the assertion made by Polig (2001) that there is no advantage in assuming that the biokinetic behaviour of the decay products is the same as that of the parent. However, the vector formulation similar to that of equation (20) was adapted to solve the independent kinetic problem but with an optimized partitioning approach of the ***A*** matrix (Fell *et al* 2007). For cases of age-dependencies of the biokinetic models, intermediate rates are found by linear interpolation for which the shared kinetic solution from *t* to *t +*d*t* is given by (Fell *et al* 2007):

\begin{equation*}{\boldsymbol{Q}}\left( {t + {\text{d}}t} \right) = {e^{{\boldsymbol{B}}\left( {\boldsymbol{t}} \right){\textbf{d}}t}}{\boldsymbol{Q}}\left( t \right){e^{{\boldsymbol{R}}{\textbf{d}}t}}.\end{equation*}

Despite the detailed approaches established in the work by Fell *et al* (2007), they commended the simplicity and effectiveness of the methodology implemented by Eckerman *et al* (1992) and iterated that the focal point may be lost if advocating for a particular approach where each approach comes with its own advantages and disadvantages. As emphasized by Fell *et al* (2007), further work to consider for an optimized biokinetic computational scheme is the use of Schur decomposition, where the biokinetic model’s matrix ***B*** is decomposed as Schur triangularization as:

\begin{equation*}{\boldsymbol{B}} = {\boldsymbol{UT}}{\boldsymbol{U}^{ - 1}}\end{equation*}

where ***T*** is an upper triangular matrix with eigenvalues in the diagonal positions and ***U*** is a unitary matrix, which in some cases where ***U*** is real, the inverse is equated to the transpose, instead of the eigenvector approach as:

\begin{equation*}{\boldsymbol{B}} = {\boldsymbol{VD}}{\boldsymbol{V}^{ - 1}}\end{equation*}

where ***D*** is diagonal containing the eigenvalues of ***B*** and ***V*** is the eigenvectors.

### 5.12. Individual monitoring for internal exposure (IMIE) (2007)

In 2007, Berkovski *et al* simultaneously developed and published a computer code called IMIE (Berkovski *et al* 2007). This code provides a set of interactive tools for the interpretation of bioassay data and assesses personalized monitoring doses. Numerical deconvolution algorithms and a library of tabulated bioassay/dose-response functions were utilized to assess an individual’s exposure to complex conditions and arbitrary intake patterns.

### 5.13. Improved dosimetry and risk assessment for plutonium-induced diseases (IMPDOS) (2008)

For case-specific exposure scenarios, IMPDOS code was developed specifically for modelling, data analysis, activity, and dose computations relying on bioassay and postmortem dataset from Mayak workers (Miller *et al* 2008). IMPDOS implemented the DLSODES in FORTRAN 77 for ODE biokinetic solving.

### 5.14. Activity and internal dose estimate (AIDE) (2008)

AIDE software is also a known software in the ID community, which was initially meant to be used as a training tool in ID. The software is programmed to estimate the activities in parts of the body classified as compartments and committed doses due to occupational exposures and for performing intake and dose estimates using bioassay data (Bertelli *et al* 2008). According to Bertelli *et al* (2008), the system of first-order DEs with constant coefficients describing the activities in compartments is solved by using the analytical computational approach of eigenvalues and eigenvectors (Bertelli and Lipsztein 1987), where the routine-based programming solving method used in AIDE has shown to be reliable for dealing with large matrices.

### 5.15. IDode (2012, 2019) and Los Alamos National Laboratory internal dose (LANL ID) (2015)

Comparable to SAAM II, IDode is an internal dose code that uses numerical solutions of ODEs defining biokinetic/physiologically-based models to estimate radiation dose (Miller *et al* 2012, 2019, Dumit *et al* 2023). IDode evolved from the predecessor RATDOSE, which was designed to evaluate data from animal experiments for investigating the efficacy of chelation agents (Miller *et al* 2012, Dumit *et al* 2020). IDode was written in Fortran with a graphical user interface (GUI) designed in Visual Basic 6 (VB6) (Miller *et al* 2019). This software utilized DLSOLDES, a Fortran differential equation solver that is proficient in solving linear and nonlinear DEs (Miller *et al* 2019). Furthermore, IDode was designed to integrate numerically evaluated forward solutions with measured data using the Bayesian method (Miller *et al* 2018, 2019) and other probabilistic models explored by Miller (2013). In addition to solving ODEs for forward models, Poudel *et al* (2018) described a discretized biokinetics method (a biokinetic model described as an interpolation table of compartmental quantities per unit intake versus time post-intake) utilizing Bayesian analysis for retrospective dosimetry. Based on the probabilistic methods described elsewhere (Miller *et al* 1999, 2000, 2001, 2002a, 2002b, 2003), a Bayesian Markov–Chain Monte Carlo ID code, also known as Los Alamos National Laboratory internal dose (LANL ID) code was developed mainly for estimation of dose from plutonium intakes (Poudel *et al* 2018). According to the study by Poudel *et al* (2018), LANL ID was revised in 2015 from FORTRAN 77 to FORTRAN 95, leveraging experience acquired at LANL.

### 5.16. J-LSODE (2019)

The ICRP has been publishing a series of recommendations for radiation protection, where the dose coefficient is known to be a quantity of relevance over the years. While the recommendations provided are comprehensive, it is relevant to note that they may not encompass all possible release or exposure scenarios and source terms in certain global regions. One such example is the case of the Japanese regulatory standards for radiation protection (Manabe *et al* 2019). The Japan Atomic Energy Agency (JAEA) was then inspired to develop a computational code for internal dosimetry based on the 2007 Recommendations of ICRP (ICRP 2007). According to Manabe *et al* (2019), LSODE (Radhakrishnan and Hindmarsh 1993) was applied to solve the ODEs for the biokinetics numerically and to compute the dose. The radiation-weighted S values were computed using piecewise cubic hermite interpolation polynomial (PCHIP) (Fritsch 1982). According to this study (Manabe *et al* 2019), no new solving methods were developed. However, to build a unified platform, the solvers (PCHIP and LSODE) were then reconstructed into Java programming language as J-LSODE and J-PCHIP, respectively, where JAEA selected Java as the programming platform due to the executability on multiple operating systems.

### 5.17. TAURUS (2020)

TAURUS, a successor of IMBA, is a new internal dose calculation software of the UK Health Security Agency (UKHSA) (Pettersson *et al* 2022). As detailed in the TAURUS information sheet from the UKHSA (UK Health Security Agency 2020), TAURUS features a GUI for the UKHSA’s internal dosimetry computer code PLEIADES, written in Fortran (Fell *et al* 2007). The methodologies employed in PLEIADES are extensively discussed in section 5.11. The TAURUS code implements the latest recommendations of the ICRP (ICRP 2007) and utilizes biokinetic and dosimetric models from the ICRP Occupational Intake of Radionuclide series of publications for calculating effective dose coefficients (Lee *et al* 2022). TAURUS serves the purpose of calculating radionuclide activity in organs and excreta of the body, as well as determining committed doses resulting from occupational exposures. According to the TAURUS information sheet from the UKHSA (UK Health Security Agency 2020), TAURUS is also capable of estimating radionuclide intakes from bioassay data using the maximum-likelihood fitting method—a methodology previously implemented in IMBA. It is important to emphasize that IMBA continues to be actively used by the internal dosimetry community. However, the distinct contribution of the TAURUS code lies in its incorporation of more recent biokinetic models for occupational intake of radionuclides.

### 5.18. IDAC-Bio (2022)

For flexibility to simulate specific exposure scenarios and intakes, a new computer code in MATLAB (IDAC-Bio) for internal dosimetry based on the new ICRP biokinetic models and specific absorbed fractions was developed (Andersson *et al* 2022). According to the developers (Andersson *et al* 2022), ICRP only publishes dose coefficients for a single acute intake of a radionuclide and for an integration period of 50 years for intake by adults and to age 70 years for intakes by pre-adults, hence, necessitating the development of the new software. Although Andersson *et al* (2022) stated that the system of equations describing the biokinetics was solved numerically, the rigor in the numerical evaluation in MATLAB was not detailed in the operational report. However, several ODE solvers are available for numerically solving different forms of ODEs, most of which have been discussed in this paper in the ODE solvers and solving method section, highlighting some applicable regimes, strengths, and weaknesses.

### 5.19. Summary

Equally significant internal dose computer codes exist. However, the selected codes presented in the discussion are based on sufficient information on ODE methods, historical usage, models implemented, accessibility, and upgrades. Internal dose computer codes such as, but not limited to, INDOS, INREM-II, and AGEDOS (Leggett *et al* 1984) were not discussed as separate subsections since many of these codes benefitted from upgrades or utilization of methodologies in currently available codes (e.g. DCAL and PLEIADES). Nonetheless, it is worth noting that these earlier codes (prior to 1998) were used for many years by the internal dosimetry community and thus had made significant contributions to computational modules used in the current era of internal dosimetry.

## 6. Conclusion

The mathematical formalisms describing biokinetic models have been introduced, underpinning a detailed review of ODE solvers, solving methods, and computational tools mainly for modeling the distribution and dosimetry of internal emitters. Additionally, the potentiality and reliability of solving the coupled system of ODEs, as in the case of biokinetic modelling, were discussed. The analysis presented herein is the first of its kind, thus providing a foundation for the comparative development of mathematical solvers and computational capabilities in the development of biokinetic modelling solvers.

In general, significant improvements made over these years, driven by the specialized community of computational dosimetry scientists focused on internal emitters for consistent optimization of computational schemes in compartmental modelling, were guided by continuously advancing methodologies for compartmental analysis with enhanced accuracy and reduced computational time. An example is the exploitation of forward models through Bayesian analysis for retrospective dosimetry (Poudel *et al* 2018). The computer codes explicitly discussed in this paper are not evidence of the authors’ approval/or endorsement for any of the programs for internal dosimetry but instead highlight the choice of computer codes and solvers applicable to fundamentally solving ODEs posed by biokinetic models/compartmental models. Additionally, it is worthwhile to remind the reader that other equally significant internal dose computer programs do exist. However, with limited available information regarding ODE solvers implemented in these programs, they were not explicitly covered in this review.

Finally, in order to advance the capabilities and expand the scope of biokinetic modelling, it is necessary to assess the appropriateness of various advanced ODE solvers and methodologies for enhancing dynamic biokinetic development. Furthermore, future attention will be directed towards modelling second-order systems in a modern programming language and refining the solving methods/solvers to effectively capture the intricacies of biokinetic models with second-order components.

## Data Availability

All data that support the findings of this study are included within the article (and any supplementary files).
